# Supplementary material for: Study protocol of a breathing and relaxation intervention included in antenatal education: A randomised controlled trial (BreLax study)
Source: PLoS One. 2024 Oct 8;19(10):e0308480. doi: 10.1371/journal.pone.0308480 (PMC11460687; doi:10.1371/journal.pone.0308480)
Supplement: S1 Table — (PDF) [file pone.0308480.s002.pdf]

Table 4 *Taxonomy of the behaviour change technique of the BreLax intervention*

**Nr.**	**Label**	**Implementation in BreLax**
1.1	Goal setting (behaviour)	Set individual behaviour: *Taking the time to do the exercises 3 times a week.*
1.2	Problem solving	Overcoming barriers - individual planning<br>*What do you consider to be good situations and good times for implementation?*
1.3	Goal setting (outcome)	Set individual goal(s): *I would like to be able to use BreLax adapted to my needs during labour and birth; I know a coping strategy that suits me.*
1.4	Action planning	*When and where do I practise?*
1.5	Review behaviour goal(s)	Check through the online brochure: *How do you feel about the intervention? Are the goals still right for you?*
1.6	Discrepancy between current behaviour and goal	Check through the online brochure: *How close do you feel to your goal?*
1.7	Review outcome goal(s)	Check through the online brochure: *Would you like to adjust your goals in light of the experience you have gained so far?*
1.8	Behavioural contract	Summary of the goals through the online brochure
1.9	Commitment	Through communication: *I don't give up right away. I want to achieve my goals.*
**2.**	**Feedback and monitoring**	
2.1	Monitoring of behaviour by others without feedback	During antenatal education classes
2.2	Feedback on behaviour	During antenatal education classes and through the online brochure.
2.3	Self-monitoring of behaviour	Through the online brochure
2.4	Self-monitoring of outcome(s) of behaviour	Through the online brochure
2.6	Biofeedback	Body awareness after breathing and relaxation technique and exercises
**3.**	**Social support**	
3.1	Social support (unspecified)	Support from partners and midwives
3.3	Social support (emotional)	Support from partner(s)
**4.**	**Shaping knowledge**	
4.1	Instruction on how to perform a behaviour	Will be discussed in the antenatal education classes, additionally there are brochures and the online brochure
4.2	Information about antecedents	Will be discussed in the antenatal education classes, additionally there are brochures and the online brochure
4.4	Behavioural experiments	Through various body exercises in antenatal education classes
**5.**	**Natural consequences**	
5.1	Information on health consequences	Evidence is presented in the brochure and emphasised in the classes.

5.4	Monitoring of emotional consequences	Taken in the course and in the online brochure: *How do you feel after the exercises?*
**6.**	**Comparison of behaviour**	
6.1	Demonstration of the behaviour	Instruction of the intervention in the course, in the brochure, and in the online brochure
6.2	Social comparison	Within classes; through social media.
6.3	Information about the approval of others	Within classes; through social media.
**7.**	**Associations**	
7.1	Prompts/cues	Through the online brochure
7.2	Cue signalling reward	Through the online brochure
7.7	Exposure	This topic will be addressed in classes, through the online brochure.
7.8	Associative learning	This topic will be addressed in classes, through the online brochure.
**8.**	**Repetition and Substitution**	
8.1	Behavioural practise/rehearsal	Through the online brochure and brochure
8.2	Behaviour Substitution	Through the online brochure and brochure
8.3	Habit formation	by practising regularly; Reminder via the online brochure
8.6	Generalisation of a target behaviour	by practising regularly; Reminder via the online brochure
**9.**	**Comparison of outcomes**	
9.1	Credible source	Through midwives and gynaecologists
9.2	Pros and Cons	Individual and independent evaluation
9.3	Comparative imagining of future outcomes	Evidence is presented in the brochure and emphasised in the classes.
**10.**	**Reward and threat**	
10.4	Social reward	Through partners and midwives
10.7	Self-incentive	Plan your personal reward; Reminder via the online brochure
10.8	Incentive (outcome)	Plan your personal reward; Reminder via the online brochure
10.9	Self-reward	Plan your personal reward; Reminder via the online brochure
10.10	Reward (outcome)	Body awareness; Plan personal reward;
**11.**	**Regulation**	
11.2	Reduce negative emotions	Through information in the course and in the brochure, with reminders via the online brochure.
11.3	Conserving Mental Resources	Through the online brochure
**12.**	**Antecedents**	
12.1	Restructuring the physical environment	Create an atmosphere of awareness
12.2	Restructuring the social environment	The partner is informed within the class about supportive role they should play.

12.4	Distraction	Raise awareness through information in class, in the brochure and in the online brochure; develop personal strategy
12.5	Adding objects to the environment	Raising awareness and drawing attention through information in class, in the brochure, and in the online brochure
**13.**	**Identity**	
13.1	Identification of self as role model	Plan individual birth, define important factors individually, information through classes
13.2	Framing/reframing	Through classes and the online brochure: *I support the birth process - I do something good for myself - I focus on my body.*
13.4	Valued self-identity	Through classes and the online brochure: *Reflecting on your strengths; remembering general self-efficacy; pick a specific situation that you have handled successfully.*
**14.**	**Scheduled consequences**	
14.4	Reward approximation	Through the online brochure: motivational statements
14.6	Situation-specific reward	Through the online brochure: commendatory statements
**15.**	**Self-belief**	
15.1	Verbal persuasion about capability	Through positive and appreciated communication from midwives and motivating words from partners.
15.2	Mental rehearsal of successful performance	Exercises at home; reminder through the online brochure
15.4	Self-talk	Reinforcement through reminder via the online brochure
**16.**	**Covert Learning**	
16.2	Imaginary reward	Imagine giving birth and believing that I am able to do it and that I am well prepared.
