# Supplementary figures and images for: Study protocol of a breathing and relaxation intervention included in antenatal education: A randomised controlled trial (BreLax study)
Source: PLoS One. 2024 Oct 8;19(10):e0308480. doi: 10.1371/journal.pone.0308480 (PMC11460687; doi:10.1371/journal.pone.0308480)

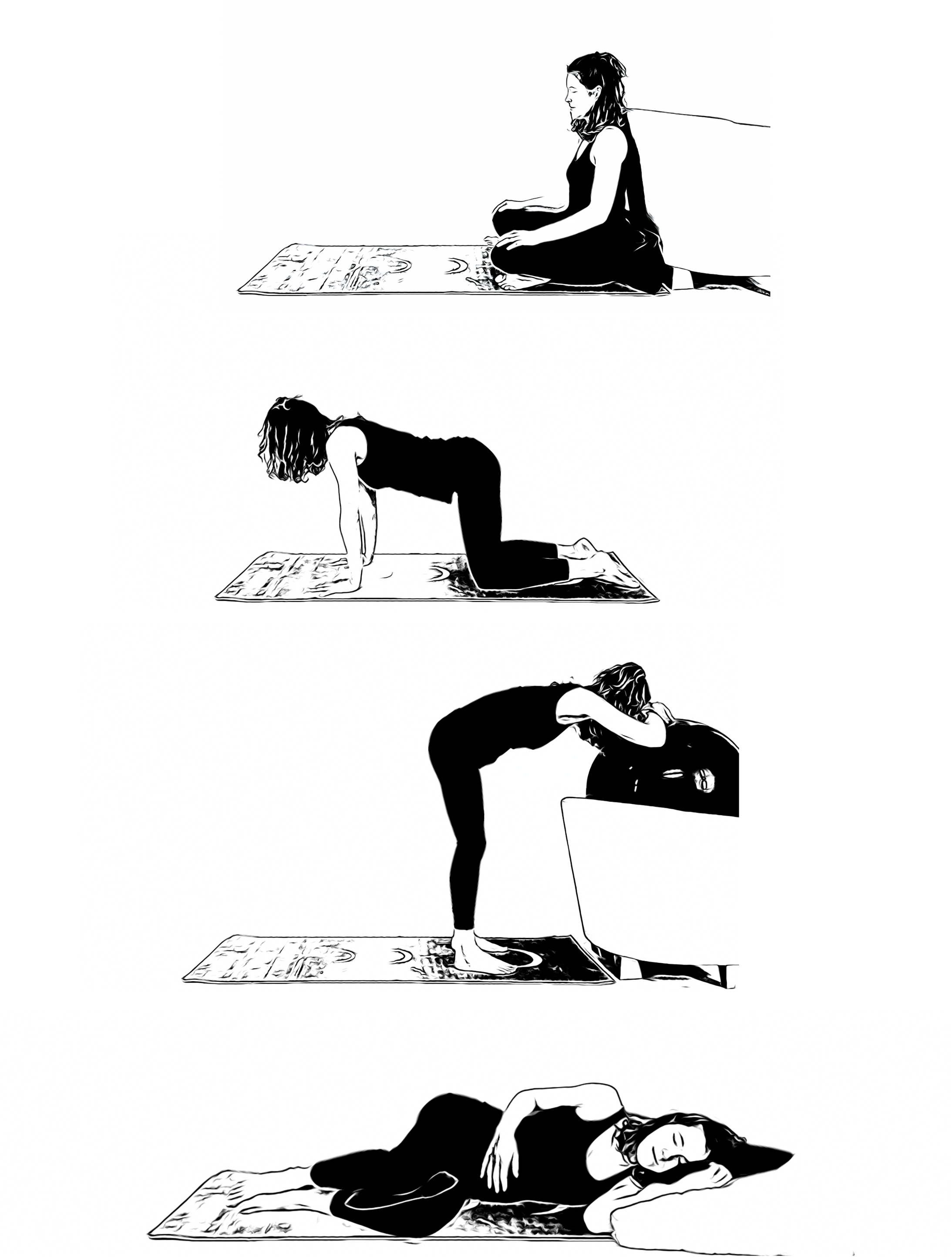

Supplement: S1 Fig — (TIF) [file pone.0308480.s003.tif]
