# Supplementary material for: Study protocol of a breathing and relaxation intervention included in antenatal education: A randomised controlled trial (BreLax study)
Source: PLoS One. 2024 Oct 8;19(10):e0308480. doi: 10.1371/journal.pone.0308480 (PMC11460687; doi:10.1371/journal.pone.0308480)
Supplement: S1 File — (PDF) [file pone.0308480.s004.pdf]

Zürcher Hochschule
für Angewandte Wissenschaften

Gesundheit

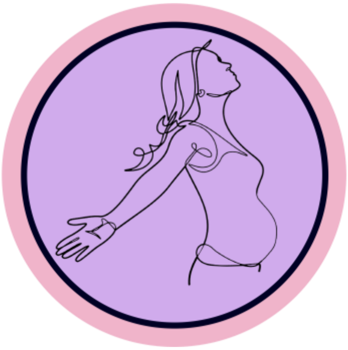

Manual for women

Effectiveness of antenatal education with an integrated Breathing and Relaxation Technique

Vanessa Leutenegger, RM, MScN, PhD Student

Forschungsstelle
Hebammen-wissenschaft

Thank you

Thank you for agreeing to take part in the BreLax study and sharing your experiences with us. By taking part, you are helping us to learn more about how you have benefited from antenatal preparation, what parts have been helpful for you during labour and what we need to work on in the future. By participating, you will enable us to examine data and potentially find positive evidence of how maternal and infant outcomes are affected by antenatal preparation with integrated breathing and relaxation techniques.

Vanessa Leutenegger

Background and aim of the study - simply explained

Today, antenatal classes are part of antenatal care in many Western countries and are offered to pregnant women and their partners in various forms (Hong et al., 2020). In Switzerland, antenatal preparation is also part of antenatal care and is co-financed by the compulsory health insurance (OKP). An antenatal education class focuses on physical and mental preparation for birth as well as important information on pain management during labour (Leutenegger et al., 2022).
Current research suggests that women who have taken part in antenatal education classes benefit from them in different ways. So far, however, we know little about which parts of antenatal preparation, the information provided or the integrated exercises, benefit women most. The aim of the study is therefore to investigate the effectiveness of an antenatal education class with an integrated breathing and relaxation technique on maternal and infant outcomes, such as self-efficacy, in comparison to a standard antenatal class.

Breathing and relaxation technique

With the help of the BreLax exercises, you will learn how to perform and utilise your breathing, muscles and relaxation. The main advantage of the exercises is that they help you to stay fit throughout your pregnancy, teach you how to breathe in a supportive way and learn how to consciously relax in as many different everyday situations as possible. For your information - breathing technique is defined as breathing with a certain number of repetitions (Evans, 2014).

Breath awareness (BA)

Conscious breathing can provide physical, mental and emotional control. Deep and conscious breathing increases blood circulation, oxygen flow and reduces stress, which benefits both you and your baby. By learning conscious breathing and relaxation techniques, you can learn to control yourself when labour starts, be aware of your pain and relax during breaks, which will boost you and your confidence (Irmak Vural & Aslan, 2019).

BreLax-Exercises

- You will get to know and use the extended exhalation in your own individual rhythm (based on the 3-6 breathing techniques for relaxation)
- You will learn and apply exercises for mental and physical relaxation
- You will learn about and apply four positions that can support you during labour (sitting supported, 4-foot position, standing supported, elevated lateral position)
- Optional: You can focus on the exercises accompanied by individual visualisation or music

The important thing is...

At this point it is important to mention that you adapt the BreLax exercises to suit you and your rhythm and use them accordingly. You will learn to use your breathing individually to help you concentrate and make your way through labour.

Breathing and relaxation techniques are only useful and helpful if you try out the BreLax exercises, practise them and can apply them in various everyday situations. Breathing and relaxation techniques can be used on any day and in any stressful or unpleasant situation (Doriana et al., 2010). For this reason, it is important that you continue to practise at home and individually. In the best case scenario, the breathing and relaxation techniques will become a kind of routine and become so internalised that you will remember the BreLax exercises during the labour, be able to recall them and apply them accordingly.

Support options

There are a number of options that can support you in the application of breathing and relaxation techniques during labour. The following factors can be taken into account when using breathing and relaxation techniques:

- Your individual prolonged exhalation, your individual breathing rhythm
- Vocalising (with an open throat) - if you feel comfortable doing so
- Mobilising the pelvis with the help of movements when it feels good for you
- The ability to recognise the difference between a state of tension and relaxation
- The ability to quickly relax tense body segments (relaxed muscle tone calms the pain signals sent to the brain, thus closing the pain control door)
- The importance of movement during labour
- Visualisation (to be decided individually)
- The involvement of a trusted companion

What can change for you when you use the breathing and relaxation techniques? The following positive changes are conceivable as a result of the breathing and relaxation techniques:

- Changed perception of pain
- The promotion of personal motivation, choice and self-determination

- The expression of your personal experience (your birth experience)
- Positive conditioning to reduce fear and pain and raise individual expectations
- Working on your individual rhythm and your active and passive attitude towards pain
- Raising awareness of pauses between contractions
- Encouraging intuitive behaviour (trust your body)
- Positive communication with your partner and/or other supportive people

Exercise instructions

Possible introductory exercise

Deep abdominal breathing

- Find a comfortable and relaxed position sitting or lying on your side.
- Place your hands on your tummy. Make contact with your baby.
- Now slowly try to let the air "flow" behind your hands into your tummy as you inhale. Your belly bulges forwards and your hands move away from each other.
- You let go and breathe out, your belly moves inwards again and your hands move closer together.

BreLax exercise

Prolonged exhalation

Procedure for an exercise session:

- You should assume an upright, supported and relaxed position, sitting, 4-foot stance, standing or in an elevated lateral position.
- Now bring your attention to your breathing and observe 5-10 normal breaths.
- Feel how your breath flows in and out of your body.
- Now deepen your breathing slightly and lengthen your exhalation individually. Possibly like this: "You breathe in for 3 bars (which you count internally) and breathe out for about twice as long (i.e. 6 bars)."
- Repeat this process for the next 10 to 12 breaths.
- Then return to your normal breathing rhythm.

Tracing

- How do you feel after the BreLax exercise?
- Feel your body, your baby, notice how your breathing has deepened and slowed down, how your body or even your pulse has changed.

Supporting positions

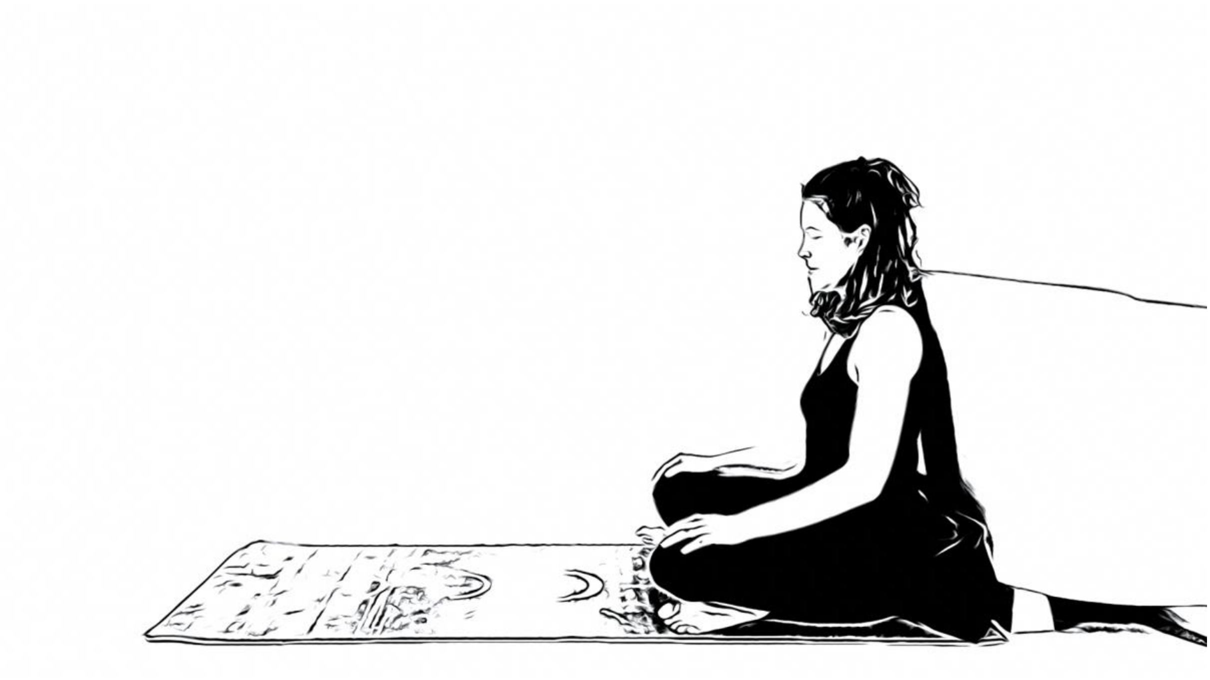

Fig. 1. Sitting supported BreLax

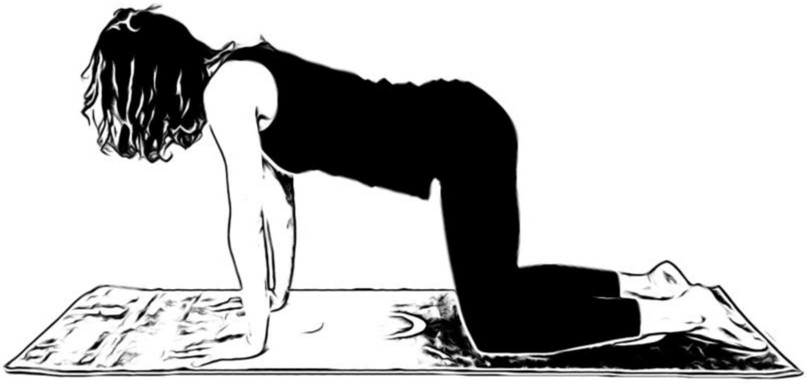

Fig. 2. 4-Point-Kneeling BreLax

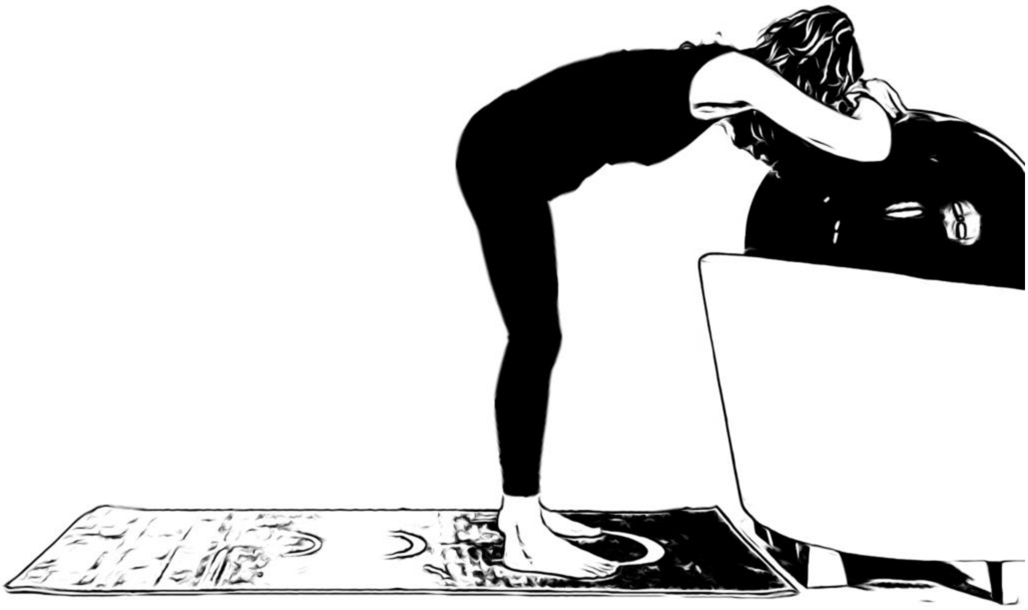

Fig. 3. Standing supported BreLax

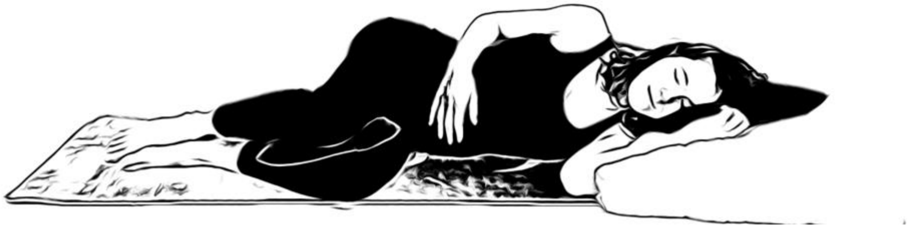

Fig. 4. Raised lateral position BreLax
