# Supplementary material for: Study protocol of a breathing and relaxation intervention included in antenatal education: A randomised controlled trial (BreLax study)
Source: PLoS One. 2024 Oct 8;19(10):e0308480. doi: 10.1371/journal.pone.0308480 (PMC11460687; doi:10.1371/journal.pone.0308480)
Supplement: S1 Data — (PDF) [file pone.0308480.s005.pdf]

# Evaluation of an antenatal education class including a breathing and relaxation technique (BreLax study)

| | |
|---|---|
| Study Type: | Other Clinical Trial according to ClinO, Chapter 4 |
| Risk Categorisation: | Category A |
| Study Registration: | 1. The study will be registered in the clinicaltrial.gov registry. The registration will be finished after the Ethical approval (Nr. )<br>2. The study will be registered in the FOPH portal SNCTP (Swiss National Clinical Trial Portal) after ethical approval |
| Sponsor-Investigator: | Vanessa Leutenegger, RM, MScN, PhD Student, Research Unit for Midwifery, Institute of Midwifery and Reproductive Health, School of Health Professions, ZHAW Zurich University of Applied Sciences, Winterthur |
| Principal Investigator | Vanessa Leutenegger, RM, MScN, PhD Student, Research Unit for Midwifery, Institute of Midwifery and Reproductive Health, School of Health Professions, ZHAW Zurich University of Applied Sciences, Winterthur |
| Investigated Intervention: | Evaluation of an antenatal education class including a breathing and relaxation technique. |
| Protocol ID | 2023 - 000183124 |
| Version and Date: | Version 2.0 10/10/23 |

**CONFIDENTIALITY STATEMENT**

The information contained in this document is confidential and the property of Vanessa Leutenegger, MScN, PhD Student. The information may not - in full or in part - be transmitted, reproduced, published, or disclosed to others than the applicable Competent Ethics Committee(s) and Regulatory Authority(ies) without prior written authorisation from the sponsor except to the extent necessary to obtain informed consent from those who will participate in the study.

## PROTOCOL SIGNATURE FORM

| | |
|---|---|
| Study Title | Evaluation of an antenatal education class including a breathing and relaxation technique (BreLax study) |
| Study ID | 2023 - 000183124 |

The Sponsor-Investigator has approved the protocol 1.3 (dated 26/07/2023) and confirmed hereby to conduct the study according to the protocol, current version of the World Medical Association Declaration of Helsinki, and ICH-GCP guidelines as well as the local legally applicable requirements.

**Sponsor-Investigator:**

Name: *Vanessa Leutenegger*

Date: 26.07.2023 ________________ Signature: [redacted] ________

**Local Project Leader at local center/site:**

Cantonal Hospital Winterthur, Clinic for Obstetrics, Brauerstrasse 15, 8400 Winterthur

Name of Local Project Leader: *Dr. med. Leila Sultan-Beyer*

Date: 11.8.23 ________________ Signature: [redacted]

## TABLE OF CONTENTS

GLOSSARY OF ABBREVATIONS 3
1 BACKGROUND AND RATIONALE 4
1.1 Introduction 4
1.2 Background 4
2 STUDY OBJECTIVES AND DESIGN 5
2.1 Hypothesis and primary objective 5
2.2 Primary and secondary endpoints 5
2.3 Study design 5
2.4 Study intervention 6
2.4.1 Breath awareness (BA) 7
2.4.2 Informational component 8
3 STUDY POPULATION AND STUDY PROCEDURES 8
3.1 Inclusion and exclusion criteria, justification of study population 8
3.2 Recruitment, screening and informed consent procedure 9
3.3 Study procedures 9
3.3.1 Potential bias and measures, to reduce them: 11
3.4 Withdrawal and discontinuation 12
4 STATISTICS AND METHODOLOGY 12
4.1 Statistical analysis plan and sample size calculation 12
4.2 Handling of missing data and drop-outs 13
5 REGULATORY ASPECTS AND SAFETY 13
5.1 Local regulations / Declaration of Helsinki 13
5.2 (Serious) Adverse Events and notification of safety and protective measures 13
5.3 (Periodic) safety reporting 14
5.4 Radiation 14
5.5 Pregnancy 14
5.6 Amendments 15
5.7 Notification and reporting upon completion, discontinuation or interruption of the study 15
5.8 Insurance 15
6 FURTHER ASPECTS 15
6.1 Overall ethical considerations 15
6.2 Risk-benefit assessment 16
7 QUALITY CONTROL AND DATA PROTECTION 16
7.1 Quality measures 16
7.2 Data recording and source data 17
7.3 Confidentiality and coding 17
7.4 Retention and destruction of study data and biological material 18
8 MONITORING AND REGISTRATION 18
9 FUNDING / PUBLICATION / DECLARATION OF INTEREST 18
11. REFERENCES 19
Appendix 1: Schedule of assessments 21

## GLOSSARY OF ABBREVATIONS

| | |
|---|---|
| *AE* | *Adverse Event* |
| *ASR* | *Annual Safety Repot* |
| *BASEC* | *Business Administration System for Ethical Committees* |
| *CRF* | *Case Report Form* |
| *CTCAE* | *Common Terminology Criteria for Adverse Events* |
| *FADP* | *Federal Act on Data Protection (in German: DSG, in French: LPD, in Italian: LPD)* |
| *eCRF* | *electronic Case Report Form* |
| *FOPH* | *Federal Office of Public Health* |
| *GCP* | *Good Clinical Practice* |
| *HRA* | *Human Research Act (in German: HFG, in French: LRH, in Italian: LRUm)* |
| *ICH* | *International Conference on Harmonisation* |
| *ClinO* | *Ordinance on Clinical Trials in Human Research (in German: KlinV, in French: OClin, in Italian: OSRUm)* |
| *SAE* | *Serious Adverse Event* |

# 1 BACKGROUND AND RATIONALE

## 1.1 Introduction

Antenatal education classes were developed to inform expectant mothers about pregnancy, labour and birth as well as the postpartum period with the aim of improving the pregnancy and childbirth experience (Ahldén et al., 2012; Ip et al., 2009). Conceptually, they were based, for example, on the pioneering work of Lamaze (1956) and Grantly Dick-Read (1944) (Svensson et al., 2009). Studies indicate positive emotional effects of antenatal education classes on labour and birth outcomes. This includes lower levels of fear of childbirth, lower rates of caesarean birth at the women`s request, a higher rate of spontaneous births, a stronger involvement of the partner, better chances of initiating breastfeeding, and a lower likelihood of developing depression symptoms during the postpartum period (Ip et al., 2009; Rouhe et al., 2013; Svensson et al., 2008). However, a standardized, evidence-based education program is missing. There is limited evidence on the link between birth preparation and neonatal outcomes (Shand et al., 2022).

## 1.2 Background

Today, women and their partners are offered antenatal education classes during pregnancy, which in Switzerland are partly financed by the health insurance. In most cases, the courses are offered by midwives and consist of a series of sessions starting in the second trimester of pregnancy. They are usually weekly courses over 6-10 evenings or weekend courses over two to three days. However, the content of antenatal classes vary widely, as does the focus and the start and duration of the program. The goal of the classes offered today is to increase knowledge and confidence in relation to pregnancy, labour and birth as well as the postpartum period (Ricchi et al., 2019), especially through information and preparation in managing labour pain through skilled techniques (Shand et al., 2022). Therefore, antenatal education classes, which focus on breathing and relaxation techniques, offer an optimal opportunity to prepare for labour and birth and the pain management.

Exercising breathing and relaxation techniques frequently and intensively before childbirth improves the feasibility of using them during birth. This leads to beneficial effects such as increased coping with labour pain and increased self-efficacy (Byrne et al., 2014). Women felt more in control as they were able to use the trained skills appropriate to their individual needs, which consequently increased their ability to feel in control, as well as being able to manage their own labour and birth (Howarth & Swain, 2019a). Self-efficacy is an important concept in labour and birth management when women want to feel confident that they will be able to maintain control during labour by managing their labour pain (Ip et al., 2009).

In maternity care, practices around birth preparation are based on experience, but they have hardly been systematically developed, implemented, and evaluated (Nolan, 2009). Although several studies have shown positive effects on self-efficacy, lower rates of epidural anaesthesia and the memory of labour pain (Abbasi et al., 2018; Bergström et al., 2009; Duncan et al., 2017; Howarth & Swain, 2019b; Levett et al., 2016; Miquelutti et al., 2013; Pan et al., 2019) the important role of self-efficacy in women's ability to cope with labour and birth has hardly been integrated into the development of antenatal education classes (Ip et al., 2009). Moreover, evidence of a potential relation between antenatal education classes and neonatal outcomes has been hard to identify so far (Gluck et al., 2020; Shand et al., 2022; Vanderlaan et al., 2022; Yohai et al., 2018).

The best available evidence for the impact of antenatal classes is from research with women who fear childbirth. For this group of women, a group psychoeducation course paired with skilled breathing and relaxation techniques had a positive effect on the women's pregnancy outcomes and childbirth experience (Ryding et al., 2018). Antenatal education classes focusing on breathing and relaxation techniques is thereby assumed to strengthen the resources of women with increased fear of childbirth and enable them to act competently and proactively during childbirth. In line with this argument, studies have shown that women who fear childbirth, especially first-time mothers, benefit from the relieving effects of breathing and relaxation on labour pain and

anxiety, (Salmela-Aro et al., 2012). Building on this recent evidence, we assume that antenatal education classes may also positively influence maternal and neonatal birth outcomes in women without heightened fear of childbirth. We therefore intend to conduct an antenatal education class focusing on a breathing and relaxation technique and assess the impact of such a class on self-efficacy, as well as other maternal and neonatal birth outcomes.

# 2 STUDY OBJECTIVES AND DESIGN

## 2.1 Hypothesis and primary objective

The following hypothesis was formed for the BreLax study:

- Women who attend an antenatal education class that include training in breathing and relaxation techniques show significantly higher self-efficacy rates than women in a standard antenatal education class that does not include training in breathing and relaxation techniques.

The main objective of the study is therefore to evaluate the effectiveness of antenatal education classes that include a breathing and relaxation technique, in relation to maternal and neonatal outcomes.

## 2.2 Primary and secondary endpoints

The primary objective is to compare the effects of antenatal education that includes a breathing and relaxation technique on self-efficacy before, during, and after birth to a standard antenatal education class without a focus on breathing and relaxation techniques and after birth.

In this study, self-efficacy is defined as follows. Self-efficacy is a person's own belief that he or she is able to cope with difficult situations and challenges on his or her own (Bandura, 1977). This means women are confident and believe that they can cope with birth by themselves.

The secondary objectives are:

(2) To identify the impact of BreLax on maternal outcomes: women`s satisfaction with their childbirth experience, self-control, pain management, mobility during labour, duration of labour and fetal attachment.

(3) To analyse the effects of BreLax on neonatal outcomes: 5-minute Apgar-Score and arterial umbilical cord pH.

(4) To determine women's perceptions of the acceptability and applicability of the learned breathing and relaxation techniques before and during labour.

(5) To assess the feasibility and effectiveness perceptions of the birth attending midwives regarding the intervention.

As background variables and potential covariates factors such as mother's sociodemographic data (e.g., age, family type, education, and occupation), the presence of chronic diseases as well as perinatal factors will be assessed. Correspondingly, relevant newborn indicators such as gestational age or birth weight will be measured.

## 2.3 Study design

The study will be conducted as a population-based randomised controlled trial (RCT) with a pre-post design and two parallel-arms: The intervention arm is an antenatal education class that includes a breathing and relaxation technique. The control arm is an antenatal education class that is like the one in the intervention arm but does not include a breathing and relaxation technique.

As quantitative pre-post outcome measures, mothers will fill in two validated questionnaires before and after birth:

- German version of the Childbirth Self-Efficacy Inventory (CBSEI-32) (Schmidt et al., 2016)
- German version of the Childbirth Experience Questionnaire (CEQ-2) (Pedersen et al., 2021).

Complementary, using a qualitative method approach, birth attendant midwives' perceptions on the feasibility and practicability of including the breathing and relaxation intervention in antenatal education classes will be assessed in semi-structured interviews.

## 2.4 Study intervention

It is recommended to start attending antenatal education in the second trimester. This applies to both the intervention and the control group. The women and their partners will be randomly assigned to one of two groups. One group serves as control group (standard care) and will receive a standard antenatal education class without a breathing and relaxation technique and exercises while the other group will receive antenatal education plus the designed intervention of a breathing and relaxation technique and exercises (BreLax: intervention group). Both groups receive information on labour and birth and the postpartum period.

The developed multicomponent intervention is based on the theoretical concept of Bandura `s self-efficacy theory (1977). According to Bandura (1977), self-efficacy is considered to be the expectation of whether an individual is capable of performing a particular behaviour in a given situation, which can be divided into outcome expectation and self-efficacy expectation. Outcome expectations refer to the individual's prediction of the possible outcomes of a set of behaviours. If someone predicts that a certain behaviour would lead to a certain outcome, that behaviour may be activated and chosen. Self-efficacy is primarily influenced by four sources of information, namely performance, vicarious experience, verbal persuasion and physiological and emotional states (Bandura, 1977).

According to Bandura (1997), a strong belief in one's own ability to exercise some control over one's physical condition can serve as a psychological prognostic indicator of the likely level of health functioning (Ip et al., 2009). In line with these assumptions, self-efficacy has been shown to have an important influence on how labour is perceived and physically endured (Leutenegger et al., 2022). Therefore, we assume that any strategy that reduces and/or controls distractions during birth such as emotional tension, can increase self-efficacy. Consequently, breathing and relaxation techniques are predicted to improve women's self-efficacy beliefs as they contribute to the control and reduction of distractions that hinder birth.

In order to increase women's self-efficacy beliefs that they trust themselves to be able to manage the birth process, the BreLax intervention will use all four sources of self-efficacy improving information. Women will experience that they can successfully perform the breathing and relaxation techniques and see that similar other can do it, too. They will also perceive reassuring comments from the class instructors and will feel less distractive emotions and physical arousal.

The core of the breathing technique is prolonged exhalation and with a certain number of repetitions and amplitudes (Evans, 2014). Such techniques has been taught for many years in antenatal education and are actively supported by many midwives during labour and birth. In the present study, the focus is on prolonged exhalation with an individual rhythm, which is learned and practised by the women during antenatal education. In addition, the women will be encouraged to continue practising at home with the help of a manual.

The main advantage of this technique is to be prepared for labour and birth, use it actively even in stressful situation, to learn an individual breathing pattern and to relax comfortably. Breathing technique means breathing with a certain number of repetitions and amplitudes (Evans, 2014).

### 2.4.1 Breath awareness (BA)

Breath awareness provides physical, mental and emotional control. Deep breathing increases blood circulation, oxygen flow and reduces stress, which benefits both mother and baby. By learning conscious breathing and relaxation techniques, women can learn to control themselves when uterine contractions start, control their pain and relax, which boosts their confidence (Irmak Vural & Aslan, 2019).

- Exercises to learn prolonged exhalation (following the 3-6 breathing technique for relaxation)
- Exercises for mental and physical relaxation (4 upright positions, standing, sitting, 4-foot, elevated lateral position)
- Optional: visualisation, music

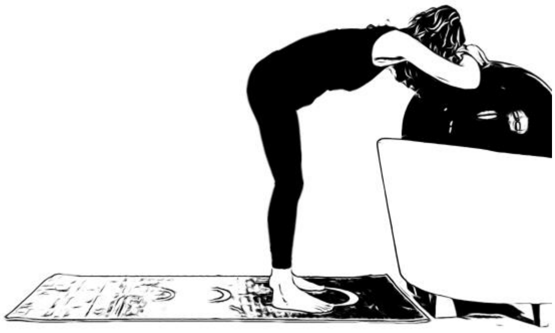

*Figure 1 Standing Position ©BreLax*

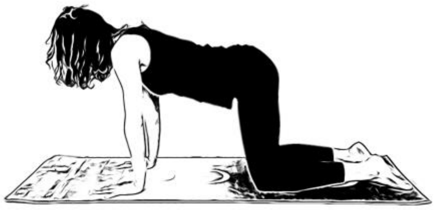

*Figure 2 4-Feet Position @BreLax*

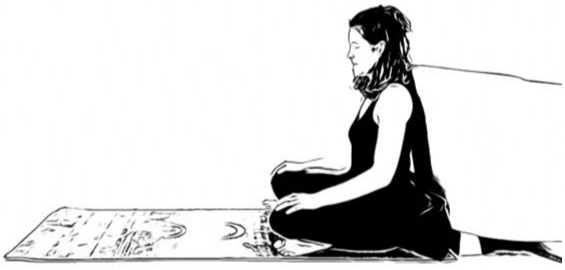

*Figure 3 Sitting Position ©BreLax*

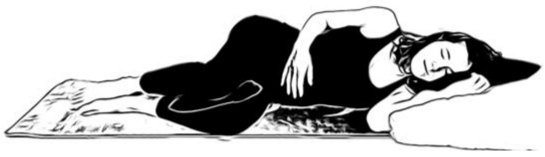

*Figure 4 Lying Position ©BreLax*

Women need to learn to use their breathing individually. Breathing and relaxation techniques are only useful and practical if women can try them out and apply them in different everyday situations. Breathing and relaxation techniques can be used on any day and in any stressful or uncomfortable situation (Doriana et al., 2010). For this reason, it is important that women continue to practice at home. In the best case, the breathing and relaxation techniques become a kind of routine or become so automated that the techniques are remembered during birth and can be used accordingly. For a kind of habit to form, it is necessary for women to practice over a period of time (Mazar & Wood, 2018). Previous literature shows that an average of 66 days is required to recall automated exercises (Gardner et al., 2020). This time period coincides optimally with the recommended start of the antenatal education class in the second trimester.

### 2.4.2 Informational component

The intervention and the standard care aim to inform women about pregnancy, labour and birth and the postpartum period. The main difference is that the intervention group focuses on the element of a breathing and relaxation technique (See Table 1). Midwives instruct the participants on how to do the exercises, how to perform the breathing patterns and how to assume the respective positions and movements. In addition, the women receive a manual to practice at home. The control group receives the standard care.

*Table 1 Content of classes*

| Content. | BreLax-Model (intervention) | Standard care (control) |
|---|---|---|
| | **Pregnancy** | |
| Nutrition | ✓ | ✓ |
| Infections | ✓ | ✓ |
| Being pregnant / Doing pregnancy | ✓ | ✓ |
| | **Labour and birth** | |
| Labour and birth | ✓ | ✓ |
| Complications | ✓ | ✓ |
| Non-pharmalogical medications | ✓ | ✓ |
| Pharmalogical medications | ✓ | ✓ |
| BreLax intervention | ✓ | x |
| Partner support | ✓ | ✓ |
| BreLax Handout | ✓ | x |
| BreLax web-application | ✓ | x |
| | **Postpartum** | |
| Baby food | ✓ | ✓ |
| Baby care | ✓ | ✓ |
| Attachment | ✓ | ✓ |
| Sleep | ✓ | ✓ |
| New Role as a mom/dad | ✓ | ✓ |

# 3 STUDY POPULATION AND STUDY PROCEDURES

## 3.1 Inclusion and exclusion criteria, justification of study population

Inclusion and exclusion criteria are defined for the randomized controlled trial as well as the individual interviews with midwives after birth.

The quantitative data collection:

- Inclusion criteria:
  - pregnant women with a singleton low-risk pregnancy
  - receiving antenatal care
  - being willing to attend an antenatal education class and planning a vaginal birth at the Canton Hospital Winterthur
  - ≥ 18 years old
  - sufficient oral and written German language knowledge

- Exclusion criteria:
    - women, who plan an elective caesarean section
    - pregnant with multiples
    - younger than 18 years old
    - do not have sufficient oral and written German language knowledge

The criteria for the individual interviews with the birth attendant midwives are that the midwife must have supported the woman for a minimum of 60 minutes and that she has sufficient written and oral German language skills.

### 3.2 Recruitment, screening and informed consent procedure

Women will be recruited via the Cantonal Hospital Winterthur and midwives in antenatal care at the hospital, surrounding gynaecological practices and midwifery practices will be informed about the study and will receive flyers for circulation in the institutions. Midwives and doctors will hand out a flyer with information about the study and the call for interested women to contact the study leader at ZHAW. Contact information of interested women will only be provided to the study leader of ZHAW after women explicitly agreed and prefer to be contacted.

The investigator will explain to each participant the nature of the study, its purpose, the procedures involved, the expected duration, the potential risks and benefits and any discomfort it may entail. Each participant will be informed that the participation in the study is voluntary and that she may withdraw from the study at any time and that withdrawal of consent will not affect her subsequent medical assistance and treatment.

The participant will be informed that her medical records about their birth will be examined by authorised individuals other than their treating obstetrician.

All participants for the study will be provided a participant information sheet and a consent form describing the study and providing sufficient information for participants to make an informed decision about their participation in the study. All potential participants will receive at least 24 hours to decide whether to participate or not.

The formal consent of a participant, using the approved consent form, will be obtained before the participant is submitted to any study procedure. The consent form will be signed and dated by the investigator simultaneously or after participants signature according to possibility. As this is a study of the risk category A and not all potential participants have scheduled visits in the hospitals, oral information can also be provided by telephone by the investigator. The study information and consent form in these cases will be sent by mail post and the participant will sign first and sent back the consent form. In order not to put pressure on the women, the investigator will only sign after the signed consent form has been returned. A copy of the signed informed consent will be given or sent to the study participant. The consent form will be retained as part of the study records. The informed consent process will be documented in the patient file and any discrepancy to the process described in the protocol will be explained.

The links with the study information to the online surveys are sent to the women by the investigator or the research assistant. The online survey will be programmed in REDCap® (Research Electronic Data Capture) and anonymised. It will enable health care providers to consent to or decline participation in the online survey itself.

Recruitment for the individual interviews with the birth attendant midwives in the Canton Hospital Winterthur will be conducted via the research assistant or the head of midwives.

### 3.3 Study procedures

The study duration will be one and a half year. The recruitment period will be one year. Participants will be recruited in their second or beginning of third trimester of pregnancy. After

signing the consent form, participants will register for an antenatal education class. Due to the course organisation at the Cantonal Hospital Winterthur, randomisation is conducted at the course level. The randomisation is performed by envelopes with the corresponding information about the course (intervention yes or no). In the randomisation process the sponsor-investigator and local project leader will be excluded.

Prior to the start of the classes, the participants fill in the online baseline questionnaire, which was sent to them by email. After the antenatal class, at around 36 weeks of gestation, the participants complete the education online questionnaire. The study assistant transfers the birth data anonymously directly from the clinic system to REDCap®. Finally, the postpartum online questionnaire is completed within four weeks after the birth (see Figure 1).

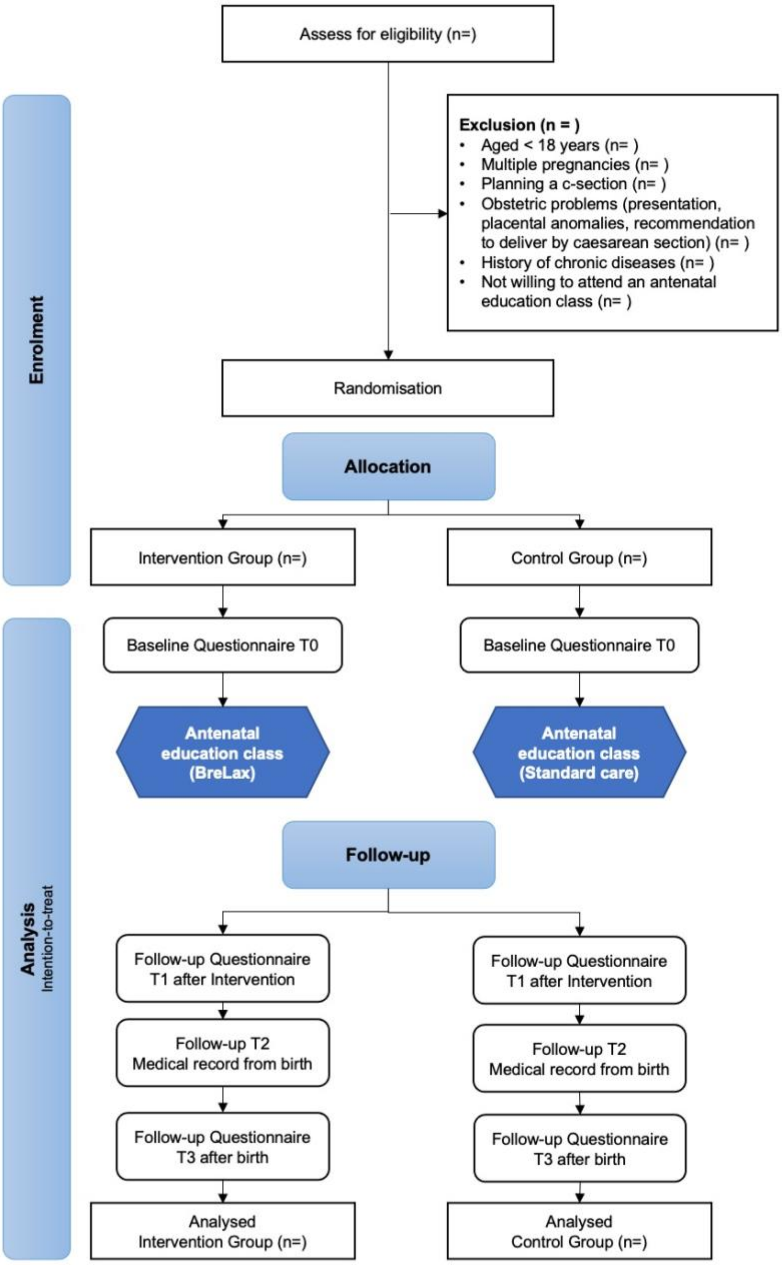


Figure 5 Study process

The following table provides an overview on the study instruments and the timing of their application for data collection:

*Table 1 Study instruments and timing of application*

| **Questionnaires / Interview** | **Time of application** | **Content** | **Filled in by** |
|---|---|---|---|
| Baseline questionnaire (T0) | After recruitment before the antenatal education class started | Socio-demographic data, medical and obstetric history, German versions of the Childbirth-Self-Efficacy Inventory | Participant |
| Education questionnaire (T1) | After intervention (around 36 weeks of gestation) | Data about the antenatal education class, German versions of the Childbirth-Self-Efficacy Inventory | Participant |
| Labour and birth questionnaire (T2) | After birth | Labour and birth outcomes for mother and child, early postpartum period | Research assistant |
| Postnatal questionnaire (T3) | Early postnatal questionnaire (within 4 weeks after birth) | German version of the Childbirth experience questionnaire, postnatal quality of life, Data about the antenatal education class | Participant |
| Semi-structured interview guide | After birth (within 10 working days) | Women's experience during birth, feasibility of the breathing and relaxation technique learned, self-efficacy and self-confidence observed | Research assistant and birth attendant midwives |

### 3.3.1 Potential bias and measures, to reduce them:

Sociodemographic questions as well as the validated questionnaires (German versions of the Childbirth-Self-Efficacy Inventory (Schmidt et al., 2016)) used for the validation of the antenatal education class will be applied before giving birth. To reduce the workload for the women, the study assistant transfers important information about the labour and birth directly from the medical birth report into the data entry tool. It is not possible to blind the midwives who conduct the antenatal education classes. However, after consent, the women are randomized and assigned to a class accordingly. In addition, the collected data will be entered by a research assistant, to ensure that data entry and data analysis are carried out by different people.

Study dropouts and possible loss to follow-up are part of research projects. For this reason, an intention-to-treat principle is conducted to minimize attrition bias.

Women after childbirth might evaluate their birth experience too optimistically. This could bias their satisfaction with the skilled breathing and relaxation technique and the attended antenatal education class. Beside questions about the antenatal education class, we will use a validated

questionnaire to assess labour and birth experience such as the childbirth experience questionnaire (Pedersen et al., 2021) to gain a more comprehensive insight. Additionally, we will use multivariable regression models to estimate the independent effect of the antenatal education class.

### 3.4 Withdrawal and discontinuation

Participation in the study is voluntary and participants can withdraw from the study without any impairment of the quality of care. Potential reasons for not completing the study process or withdrawing from the study might be that women will withdraw from the study consent or not complete the antenatal education class. We estimate not completing the class will be rare. The loss of follow-up was considered in the sample calculation. Sociodemographic and pregnancy-related characteristics, which were collected before these participants dropped out from the study, will be compared with those completing the whole study. This will provide information, if systematic patterns of dropping outs can be observed and inform the generalisability of the results. The comparison might also give information, if inclusion and exclusion criteria should be re-evaluated in follow-up studies.

Data will be entered into REDCap®. The data base in REDCap® will be programmed at ZHAW and will enable data entry in the Canton Hospital Winterthur and data download by the sponsor-investigator at ZHAW to download it in different formats. Data will then be stored on a secured pool at ZHAW. Data is stored on a dedicated, encrypted project file share provided by ZHAW's IT department. Access is limited to project members (restricted access by IT) and requires ZHAW-managed user accounts. Password policies are enforced, logins are monitored and logged. File access is logged and auditing enabled. External access requires two-factor authentication (SMS token). Data is physically stored on centralized storage, with data redundancy across ZHAW's two data-centres, daily full backups and anti-ransomware measures in place. A centralized anti-malware solution continuously scans files for malicious content. Storage capacity is up to 500 GB. Individual files/folders can be restored by users from hourly (up to 2 days), daily (up to 9 days) and weekly snapshots (up to two months).

The participant identification list, which links the study numbers to personal details of women will be stored in a separate secured pool. If participants will withdraw from the study, their study key will be destroyed deleting all personal information from the study list and their anonymised data will be used for analyses that are possible with it.

## 4 STATISTICS AND METHODOLOGY

### 4.1 Statistical analysis plan and sample size calculation

The socio-demographic and obstetric characteristics will be described employing descriptive statistics including frequency and percentage, mean and standard deviation, median and percentiles. The primary outcomes and secondary maternal and neonatal variables like women`s satisfaction with their childbirth experience, self-confidence, pain management, mobility during labour, duration of labour, bonding, 5-minute Apgar-Score and umbilical cord pH will be compared between the study groups using repeated-measures ANOVAs and generalized estimating equations and mixed effects models. Quantitative data is analysed using R. The significance level will be two-sided $\alpha = 0.05$.

The sample size in the intervention phase was calculated based on the scores for the variable of childbirth self-efficacy using G-Power software. Based on the results of Ip et al. (2009) on the effectiveness of an antenatal education class (multicomponent intervention) to increase women's self-efficacy during labour and considering M1= 121.70 of the intervention group (the mean score for childbirth self-efficacy inventory (CBSEI) from the subscale efficacy expectancy (EE-16) posttest at 37th week, SD1 = 23.64, M2 = 98.46 (the mean score for CBSEI from the subscale EE-16), SD2 = 23.36 in the control group, two sided $\alpha$ =0.05, and power = 0.80 the mean (SD) posttest EE-16 at 37th week were 119.22 (23.64) for the intervention and 98.46 (23.36) for the

control group the calculated number of participants for each group will be 26. Assuming an attrition rate of 10%, the final sample size will be 29 in each group and 58 in total.

The qualitative data collected from the attendant birth midwives will be conducted by a semi-structured interview guide. The data will be transcribed verbatim and analysed using content analysis (Kuckartz & Rädiker, 2022). The total target sample size for the individual interviews with midwives is 12-15. Variation enriches the results and can be achieved with 12 to 15 interviews. This is important to provide a good basis for the evaluation of the antenatal education class and the feasibility as well as the practicability of the skilled breathing and relaxation technique.
Qualitative data will be analysed using MAXQDA. Any deviation from the original statistical plan will be described and justified in the final trial report.

### 4.2 Handling of missing data and drop-outs

The data will be planned and analysed using the intent-to-treat principle (ITT). If missing data is >5%, the possibility of multiple imputation will be examined. We intend to minimise missing data and will for example design mandatory questions in the questionnaires and send reminders to participants to complete the follow-up questionnaires.

The dropout rate is estimated at about 10 % and was taken into account accordingly in the sample size calculation. Recruitment has to take place during pregnancy in order to be able to observe the evaluation of the intervention. In obstetrics, it is never possible to predict how the birth will proceed and how the childbirth experience will affect the participants. Therefore, loss of follow-up for medical reasons cannot be avoided. The research assistants as well as the lead midwife will monitor the study process on site and remind the participants to fill in the questionnaires.

## 5 REGULATORY ASPECTS AND SAFETY

### 5.1 Local regulations / Declaration of Helsinki

This study is conducted in compliance with the protocol, the current version of the Declaration of Helsinki, the ICH-GCP the HRA as well as other locally relevant legal and regulatory requirements.

### 5.2 (Serious) Adverse Events and notification of safety and protective measures

An Adverse Event (AE) is any untoward medical occurrence in a patient or a clinical investigation subject which does not necessarily have a causal relationship with the trial procedure. An AE can therefore be any unfavourable or unintended finding, symptom, or disease temporally associated with a trial procedure, whether or not related to it.

A Serious Adverse Event (SAE) (ClinO, Art. 63) is any untoward medical occurrence that

- Results in death or is life-threatening,
- Requires in-patient hospitalisation or prolongation of existing hospitalisation,
- Results in persistent or significant disability or incapacity, or
- Causes a congenital anomaly or birth defect

Both Investigator and Sponsor-Investigator make a causality assessment of the event to the trial intervention, (see table below based on the terms given in ICH E2A guidelines). Any event assessed as possibly, probably or definitely related is classified as related to the trial intervention.

| Relationship | Description |
|---|---|
| Definitely | Temporal relationship<br>Improvement after dechallenge*<br>Recurrence after rechallenge<br>(or other proof of drug cause) |
| Probably | Temporal relationship<br>Improvement after dechallenge<br>No other cause evident |
| Possibly | Temporal relationship<br>Other cause possible |
| Unlikely | Any assessable reaction that does not fulfil the above conditions |
| Not related | Causal relationship can be ruled out |
| *Improvement after dechallenge only taken into consideration, if applicable to reaction | |

Both Investigator and Sponsor-Investigator make a severity assessment of the event as mild, moderate or severe. Mild means the complication is tolerable, moderate means it interferes with daily activities and severe means it renders daily activities impossible.

**Reporting of SAEs** (see ClinO, Art. 63)

All SAEs are documented and reported immediately (within a maximum of 24 hours) to the Sponsor-Investigator of the study.

If it cannot be excluded that the SAE occurring in Switzerland is attributable to the intervention under investigation, the Investigator reports it to the Ethics Committee via BASEC within 15 days.

**Follow up of (Serious) Adverse Events**

Participants, who experience an (Serious) Adverse Event during childbirth will be contacted after terminating the study to answer questions, to ensure . If necessary, contact details of professionals, who could offer additional support, will be provided.

**Notification of safety and protective measures** (see ClinO, Art 62, b)

If immediate safety and protective measures have to be taken during the conduct of the study, the investigator notifies the Ethics committee of these measures, and of the circumstances necessitating them, within 7 days.

## 5.3 (Periodic) safety reporting

An annual safety report (ASR) is submitted once a year to the local Ethics Committee by the Investigator (ClinO, Art. 43 Abs 1).

## 5.4 Radiation

Not applicable

## 5.5 Pregnancy

All participants will be pregnant because of the nature of the study. Reporting of pregnancies will therefore not be necessary.

### 5.6 Amendments

Substantial changes to the study setup and study organization, the protocol and relevant study documents are submitted to the Ethics Committee for approval before implementation. Under emergency circumstances, deviations from the protocol to protect the rights, safety and well-being of human subjects may proceed without prior approval of the Ethics Committee. Such deviations shall be documented and reported to the Ethics Committee as soon as possible.

Substantial amendments are changes that affect the safety, health, rights and obligations of participants, changes in the protocol that affect study objective(s) or central research topic, changes of study site(s) or of study leader and sponsor (ClinO, Art. 29).

A list of substantial changes is also available on www.swissethics.ch.

A list of all non-substantial amendments will be submitted once a year to the competent EC together with the ASR.

### 5.7 Notification and reporting upon completion, discontinuation or interruption of the study

The Sponsor-Investigator may terminate the study prematurely according to certain circumstances, e.g.

- Ethical concerns,
- Insufficient participant recruitment,
- When the safety of the participants is doubtful or at risk (e.g. when the benefit-risk assessment is no longer positive),
- Alterations in accepted clinical practice that make the continuation of the study unwise, or
- Early evidence of harm or benefit of the experimental intervention

Upon regular study completion, the Ethics Committee is notified via BASEC within 90 days (ClinO, Art. 38).

Upon premature study termination or study interruption, the Ethics Committee is notified via BASEC within 15 days (ClinO, Art. 38).

A final report is submitted to the Ethics Committee via BASEC within a year after completion or discontinuation of the study, unless a longer period is specified in the protocol (ClinO, Art. 38).

### 5.8 Insurance

In the event of study-related damage or injuries, the liability of Zurich University of Applied Sciences provides compensation, except for claims that arise from misconduct or gross negligence. ZHAW has a liability insurance with an overall sum insured over CHF ten Mio. With one Mio. per participants to cover personal injuries. The study is registered correctly with Zurich Insurance.

## 6 FURTHER ASPECTS

### 6.1 Overall ethical considerations

Health-related data is considered to be sensitive personal data by the Federal Act on Data Protection. Thus, procedures must comply with the current GCP standards and the Human Research Act of Switzerland. Throughout the research process, we will pay close attention to dynamic ethical issues. We consider general ethical issues when designing, implementing, and reporting our research (e.g., following publication rules).

In the study information and the consent form, the study and its purpose will be described, and it will be highlighted that participation is entirely voluntary and that participation can be refused or withdraw at any stage, with no further consequences. Furthermore, participants will be informed about the intended research project and all benefits and risks (principle of autonomy). All data will be encoded which makes it impossible to conclude on a specific data without the participant identification list. The participant identification list will only be accessible to a sub-group of the study personnel and only people with appropriate training in Good Clinical Practice (GCP). Data will be threated confidentially. We conclude that our research is in the interest of the childbearing women (principle of fairness or justice), and that the women could directly benefit from the antenatal education class and therefore from the research (principle of beneficence). In order to minimize outside influence and manipulation, we openly account for our purposes and methods.

We will validate an antenatal education class including a breathing and relaxation technique. The planned study consists of the validation and evaluation of an antenatal education class to increase women`s self-confidence as well as their self-efficacy. The entire project of development, evaluation and implementation is thought through and elaborated with the help of the MRC Framework (Michie et al., 2008, 2011).

Finally, we will seek ethical approval for the evaluation of the antenatal education class including a breathing and relaxation technique to increase women`s self-efficacy at the Ethics Committee of the Canton Zurich.

### 6.2 Risk-benefit assessment

We do not expect any serious risks for the participants. Antenatal education is part of antenatal care and is financed by the obligatory health insurance (OKP). Every woman has the opportunity to attend an antenatal education class if she would like to. Women benefit from the information provided in the antenatal class as well as the exercises to support them during the labour and birth. However, participants will complete questionnaires during pregnancy and after birth. This could be an additional challenge and a lager workload for women. In addition, it is possible that the childbirth experience will become present through the questionnaires. This is why the participants are offered the opportunity to get in touch with the supervising midwife and to have a follow-up talk with the birth attendant midwife. If further support is indicated, it is possible to involve the relevant specialists at the Cantonal Hospital Winterthur.

## 7 QUALITY CONTROL AND DATA PROTECTION

### 7.1 Quality measures

Several quality measures will be installed:

- The antenatal midwives in the Canton Hospital Winterthur will be trained before the intervention. The investigator organises a 2-hour workshop where the course concept as well as the breathing and relaxation technique is discussed and practised together. In addition, the investigator is always available to answer questions from the midwives. Midwives receive a handout with the background and the breathing and relaxation technique.
- Data will be collected using REDCap®. REDCap® is a secure web application for building and managing surveys as well as databases compliant to the demands of GCP Swiss regulations regarding clinical studies. An audit trail will document all changes made to the data. The audit trail contains the person that made the changes, the time the change was made, and the data before the change. Automated checks implemented in the data entry field further check the data for plausibility and completeness as it is saved. The software gives continuous feedback to the users as

how to correct the data in order to comply to defined rules. Erroneous and missing data are marked for follow up.
- The investigator at ZHAW will be responsible for data checking and data storing using REDCap® correctly.

For quality assurance the sponsor, the Ethics Committee or an independent trial monitor may visit the research sites. Direct access to the source data and all study related files is granted on such occasions. All involved parties keep the participant data strictly confidential.

### 7.2 Data recording and source data

Quantitative data (questionnaire data, labour and birth data from medical records) will be entered by a midwife and a research assistant in the study site to the open source software REDCap® (Research Electronic Data Capture). REDCap® is an international commercial study software compliant to the demands of the current GCP standards and the Human Research Act of Switzerland (https://www.project-redcap.org/software/). REDCap® is a secure web application for building and managing surveys as well as databases compliant to the demands of GCP Swiss regulations regarding clinical studies. Data can be entered from any computer connected to the internet providing current browser software (e.g., Mozilla Firefox). Data entry fields in REDCap® are in a specific format that allows only entering data in this format (e.g., date fields, number fields). An audit trail will document all changes made to the data. The audit trail contains the person that made the changes, the time the change was made, and the data before the change. Automated checks implemented in the data entry field further check the data for plausibility and completeness as it is saved. The software gives continuous feedback to the users as how to correct the data in order to comply to defined rules. Erroneous and missing data are marked for follow up. The database will contain a CRF for each participant with a unique study ID and the year of birth. It will not contain any further personal data such as name or birth date.

We plan to provide a protocol with detailed descriptions on how the data was recorded. The metadata will include details about the date and time of data collection, data processing methods, analytical steps, units of measurements, and identifier on the person performing processing steps. This information will be stored in a README-file for each dataset.

### 7.3 Confidentiality and coding

Trial and participant data will be handled with uttermost discretion and is only accessible to authorised personnel who require the data to fulfil their duties within the scope of the study. On the CRFs and other study specific documents, participants are only identified by a unique participant number. The participant identification list will be stored on a separate secured pool on the server of ZHAW. All data will be encoded which makes it impossible to conclude on a specific data without the participant identification list. The participant identification list will only be accessible to a sub-group of the study personnel and only people with appropriate training in Good Clinical Practice (GCP).

The participant identification list is stored in the study centre in password protected pool or closed locker.

Only non-genetic data is used.

Biological material: not applicable

### 7.4 Retention and destruction of study data and biological material

All data will be stored for a minimum of ten years on restricted servers at ZHAW and made available under the respective terms of usage upon request.

## 8 MONITORING AND REGISTRATION

A researcher from ZHAW with a GCP course, who is not involved in the project, will conduct the monitoring visits. Monitoring visits are planned twice during the one-year data collection period. During these visits, data entry of labour and birth data will be checked. A total of 10% of labour and birth data, including those of the first recruited woman will be checked comparing with medical records. If error rates are above 5%, all labour and birth data of this site needs to be checked. The study sites will make source data/documents accessible to the monitors and questions are answered during monitoring.

The study will be registered in the International Clinical Trials Registry Platform (ICTRP). Additionally, it is registered in the Swiss National Clinical Trial Portal (SNCTP) via BASEC with the Ethical proposal.

## 9 FUNDING / PUBLICATION / DECLARATION OF INTEREST

The study is part of a PhD project and is therefore supported by both the Zurich University of Applied Science and the University of Zurich.

The study is funded by the Amaari Foundation. The Contract was signed with the Canton Hospital Winterthur which regulate the collaboration, the rights regarding the data and the publication policy of the study. Results of the study will be published in international peer-reviewed scientific journals as well as in professional journals and will be presented at national and international conferences. We will follow the recommendation of the Academies of Sciences and Humanities Switzerland.

## 11. REFERENCES

Abbasi, P., Charandabi, S. M.-A., & Mirghafourvand, M. (2018). Comparing the effect of e-learning and educational booklet on the childbirth self-efficacy: A randomized controlled clinical trial. *The Journal of Maternal-Fetal & Neonatal Medicine*, *31*(5), 644–650. https://doi.org/10.1080/14767058.2017.1293031

Ahldén, I., Ahlehagen, S., Dahlgren, L. O., & Josefsson, A. (2012). Parents' Expectations About Participating in Antenatal Parenthood Education Classes. *The Journal of Perinatal Education*, *21*(1), 11–17. https://doi.org/10.1891/1058-1243.21.1.11

Bandura, A. (1977). Self-efficacy: Toward a unifying theory of behavioral change. *Psychological Review*, *84*(2), 191–215. https://doi.org/10.1037/0033-295X.84.2.191

Bergström, M., Kieler, H., & Waldenström, U. (2009). Effects of natural childbirth preparation versus standard antenatal education on epidural rates, experience of childbirth and parental stress in mothers and fathers: A randomised controlled multicentre trial. *BJOG: An International Journal of Obstetrics & Gynaecology*, *116*(9), 1167–1176. https://doi.org/10.1111/j.1471-0528.2009.02144.x

Byrne, J., Hauck, Y., Fisher, C., Bayes, S., & Schutze, R. (2014). Effectiveness of a Mindfulness-Based Childbirth Education Pilot Study on Maternal Self-Efficacy and Fear of Childbirth. *Journal of Midwifery & Women's Health*, *59*(2), 192–197. https://doi.org/10.1111/jmwh.12075

Duncan, L. G., Cohn, M. A., Chao, M. T., Cook, J. G., Riccobono, J., & Bardacke, N. (2017). Benefits of preparing for childbirth with mindfulness training: A randomized controlled trial with active comparison. *BMC Pregnancy and Childbirth*, *17*(1), 140. https://doi.org/10.1186/s12884-017-1319-3

Evans, E. (2014). *The Role of Yoga: Breathing, Meditation and Optimal Fetal Positioning*. 4.

Gluck, O., Pinchas-Cohen, T., Hiaev, Z., Rubinstein, H., Bar, J., & Kovo, M. (2020). The impact of childbirth education classes on delivery outcome. *International Journal of Gynecology & Obstetrics*, *148*(3), 300–304. https://doi.org/10.1002/ijgo.13016

Howarth, A. M., & Swain, N. R. (2019a). Skills-based childbirth preparation increases childbirth self-efficacy for first time mothers. *Midwifery*, *70*, 100–105. https://doi.org/10.1016/j.midw.2018.12.017

Howarth, A. M., & Swain, N. R. (2019b). Skills-based childbirth preparation increases childbirth self-efficacy for first time mothers. *Midwifery*, *70*, 100–105. https://doi.org/10.1016/j.midw.2018.12.017

Ip, W.-Y., Tang, C. S., & Goggins, W. B. (2009). An educational intervention to improve women's ability to cope with childbirth. *Journal of Clinical Nursing*, *18*(15), 2125–2135. https://doi.org/10.1111/j.1365-2702.2008.02720.x

Irmak Vural, P., & Aslan, E. (2019). Emotional freedom techniques and breathing awareness to reduce childbirth fear: A randomized controlled study. *Complementary Therapies in Clinical Practice*, *35*, 224–231. https://doi.org/10.1016/j.ctcp.2019.02.011

Kuckartz, U., & Rädiker, S. (2022). *Qualitative Inhaltsanalyse: Methoden, Praxis, Computerunterstützung: Grundlagentexte Methoden* (5. Auflage). Beltz Juventa.

Leutenegger, V., Grylka-Baeschlin, S., Wieber, F., Daly, D., & Pehlke-Milde, J. (2022). The effectiveness of skilled breathing and relaxation techniques during antenatal education on maternal and neonatal outcomes: A systematic review. *BMC Pregnancy and Childbirth*, *22*(1), 856. https://doi.org/10.1186/s12884-022-05178-w

Levett, K. M., Smith, C. A., Bensoussan, A., & Dahlen, H. G. (2016). Complementary therapies for labour and birth study: A randomised controlled trial of antenatal integrative medicine for pain management in labour. *BMJ Open*, *6*(7), e010691. https://doi.org/10.1136/bmjopen-2015-010691

Michie, S., Abraham, C., Eccles, M. P., Francis, J. J., Hardeman, W., & Johnston, M. (2011). Strengthening evaluation and implementation by specifying components of behaviour change interventions: A study protocol. *Implementation Science*, *6*, 10. https://doi.org/10.1186/1748-

5908-6-10

Michie, S., Johnston, M., Francis, J., Hardeman, W., & Eccles, M. (2008). From Theory to Intervention: Mapping Theoretically Derived Behavioural Determinants to Behaviour Change Techniques. *Applied Psychology*, *57*(4), 660–680. https://doi.org/10.1111/j.1464-0597.2008.00341.x

Miquelutti, M. A., Cecatti, J. G., & Makuch, M. Y. (2013). Evaluation of a birth preparation program on lumbopelvic pain, urinary incontinence, anxiety and exercise: A randomized controlled trial. *BMC Pregnancy and Childbirth*, *13*(1), 154. https://doi.org/10.1186/1471-2393-13-154

Nolan, M. L. (2009). Information Giving and Education in Pregnancy: A Review of Qualitative Studies. *The Journal of Perinatal Education*, *18*(4), 21–30. https://doi.org/10.1624/105812409X474681

Pan, W.-L., Gau, M.-L., Lee, T.-Y., Jou, H.-J., Liu, C.-Y., & Wen, T.-K. (2019). Mindfulness-based programme on the psychological health of pregnant women. *Women and Birth*, *32*(1), e102–e109. https://doi.org/10.1016/j.wombi.2018.04.018

Pedersen, A., Sieprath, K., & Köhler, M. (2021). Validierung der deutschen Übersetzung des Childbirth Experience Questionnaire (CEQ2). *Diagnostica*, *67*(2), 87–97. https://doi.org/10.1026/0012-1924/a000267

Ricchi, A., Corte, S. L., Molinazzi, M. T., Messina, M. P., Banchelli, F., & Neri, I. (2019). Study of childbirth education classes and evaluation of their effectiveness. *La Clinica Terapeutica*, *1*(171), Article 171.

Rouhe, H., Salmela-Aro, K., Toivanen, R., Tokola, M., Halmesmäki, E., & Saisto, T. (2013). Obstetric outcome after intervention for severe fear of childbirth in nulliparous women – randomised trial. *BJOG: An International Journal of Obstetrics & Gynaecology*, *120*(1), 75–84. https://doi.org/10.1111/1471-0528.12011

Ryding, E. L., Read, S., Rouhe, H., Halmesmäki, E., Salmela‐Aro, K., Toivanen, R., Tokola, M., & Saisto, T. (2018). Partners of nulliparous women with severe fear of childbirth: A longitudinal study of psychological well-being. *Birth*, *45*(1), 88–93. https://doi.org/10.1111/birt.12309

Salmela-Aro, K., Read, S., Rouhe, H., Halmesmäki, E., Toivanen, R. M., Tokola, M. I., & Saisto, T. (2012). Promoting positive motherhood among nulliparous pregnant women with an intense fear of childbirth: RCT intervention. *Journal of Health Psychology*, *17*(4), 520–534. https://doi.org/10.1177/1359105311421050

Schmidt, G., Stoll, K., Jäger, B., & Gross, M. M. (2016). [German Version of the Childbirth Self-Efficacy Inventory and its Short Form]. *Zeitschrift Fur Geburtshilfe Und Neonatologie*, *220*(1), 28–34. https://doi.org/10.1055/s-0035-1547296

Shand, A. W., Lewis-Jones, B., Nielsen, T., Svensson, J., Lainchbury, A., Henry, A., & Nassar, N. (2022). Birth outcomes by type of attendance at antenatal education: An observational study. *Australian and New Zealand Journal of Obstetrics and Gynaecology*, *n/a*(n/a). https://doi.org/10.1111/ajo.13541

Svensson, J., Barclay, L., & Cooke, M. (2008). Effective Antenatal Education: Strategies Recommended by Expectant and New Parents. *The Journal of Perinatal Education*, *17*(4), 33–42. https://doi.org/10.1624/105812408X364152

Svensson, J., Barclay, L., & Cooke, M. (2009). Randomised-controlled trial of two antenatal education programmes. *Midwifery*, *25*(2), 114–125. https://doi.org/10.1016/j.midw.2006.12.012

Vanderlaan, J., Gatlin, T., & Shen, J. (2022). Outcomes of Childbirth Education in PRAMS, Phase 8. *Maternal and Child Health Journal*. https://doi.org/10.1007/s10995-022-03494-3

Yohai, D., Alharar, D., Cohen, R., Kaltian, Z., Aricha-Tamir, B., Aion, S. B., Yohai, Z., & Weintraub, A. Y. (2018). The effect of attending a prenatal childbirth preparedness course on labor duration and outcomes. *Journal of Perinatal Medicine*, *46*(1), 47–52. https://doi.org/10.1515/jpm-2016-0345

## Appendix 1: Schedule of assessments

| **Time (hour, day, week)** | **>-1 day** | > 12 weeks of gestation | 2nd trimester | After intervention (~36 weeks gestation) | Birth records | Postnatal (4 to 8 weeks after birth) |
|---|---|---|---|---|---|---|
| **Visit/Activity** | Information (visit or telephone) | Screening (face-to-face or mail post) | Baseline online survey | Intervention online survey | Maternal and neonatal brith data | Postnatal online survey |
| **Oral and written patient information** | + | | | | | |
| **Written consent** | | + | | | | |
| **Inclusion-/ exclusion criteria** | + | + | | | | |
| **Medical history** | | | + | | + | |
| **Physical examination** | | | | | | |
| **Participant characteristics** | | | + | | + | |
| **Intervention** | | | | | + | |
| **Questionnaire** | | | + | | + | + |
| **Sampling** | | | | | | |
| **Safety** | | + | | | + | |
