# Supplementary material for: Study protocol of a breathing and relaxation intervention included in antenatal education: A randomised controlled trial (BreLax study)
Source: PLoS One. 2024 Oct 8;19(10):e0308480. doi: 10.1371/journal.pone.0308480 (PMC11460687; doi:10.1371/journal.pone.0308480)
Supplement: Attachment — Submitted filename: Referee report for Brelax study.pdf [file pone.0308480.s006.pdf]

Referee's report for 'Study protocol of a breathing and relaxation intervention included in antenatal education: a randomized controlled trial (BreLax study).

The paper is quite well written and well organized. I confine my comments to statistical matters.

L174: Why verify eligibility by phone? Are there no baseline (clinical) variables to be collected at the onset? Or values of baseline variables have to meet certain threshold before admission to the trial? If so, eligibility can be confirmed on site during the baseline interview.

L180: What is the interpretation of an effect size of 0.75 in the context of the trial.

L183: The 20% attrition rate comes from nowhere without justifications. This results in a sample size of of 35 subjects to be recruited for each arm. The hospital has 1800 births and there was a brief discussion of contamination. So why not perform the trial staggered over time to minimize contamination?

L189: Why have the midwife use envelopes to randomize subjects into group membership. It is the same reason why you don't want the same statistician to randomize the trial and perform the analysis.

L334 'the possibility of multiple imputation' will be examined. This is very vague. Please elaborate. Identify situations if and when you will use multiple imputation.

L344 You also reference doing the analyses using a serious of linear mixed effects models. What models do you have in mind? Please provide more details and display the regression models and show what are the random and fixed effects for added clarity

Further comments.

Throughout, the methodology seems to assume the outcome is normally distributed. Where are the checks and what is the proposed methodology if they are not normally distributed?
